# Sustainable Approaches to Selective Conversion of Cellulose Into 5-Hydroxymethylfurfural Promoted by Heterogeneous Acid Catalysts: A Review

**DOI:** 10.3389/fchem.2022.880603

**Published:** 2022-05-10

**Authors:** Yuanyong Yao, Shixue Chen, Meng Zhang

**Affiliations:** School of Material and Chemical Engineering, Tongren University, Tongren, China

**Keywords:** 5-Hydroxymethylfurfural, cellulose, biomass resources, solid acids, selective conversion, heterogeneous acid catalysis

## Abstract

5-Hydroxymethylfurfural (5-HMF) as a triply catalytic product is a value-added refining chemical in industry production. 5-HMF as biomass feedstock enables to be transformed into other high-value industrial compounds, such as 2,5-furandicarboxylic acid (FDCA), 5-hydroxymethyl-2-furancarboxylic acid (HMFCA), 5-formyl-2-furancarboxylic acid (FFCA), 2,5-diformylfuran (DFF), 2,5-bis(aminomethyl)furan (BAMF), and 2,5-dimethylfuran (DMF). Hence, catalytic conversion of biomass into 5-HMF has been given much more attention by chemists. In this review, some latest studies about the conversion of cellulose to 5-HMF have been introduced systematically. Solid acids such as heterogeneous catalysts have been widely applied in the conversion of cellulose into 5-HMF. Therefore, some novel solid acids with Brønsted and/or Lewis acidic sites, such as sulfonated solid acids, carbon-based acids, and zeolite particles employed for biomass conversions are listed.

## 1 Introduction

**FIGURE 1:**
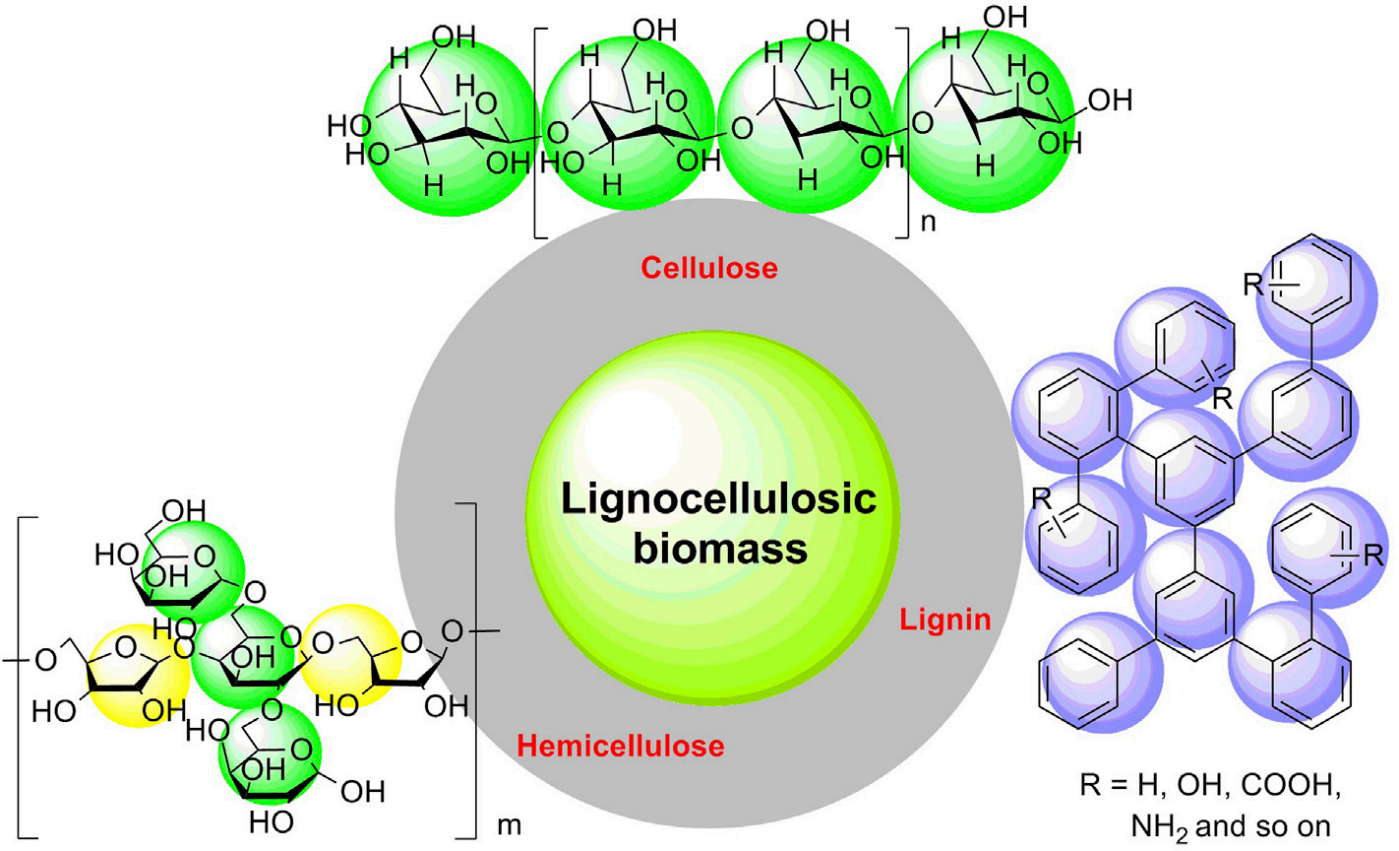
Compositions of lignocellulosic biomass.

With the progressive increase in consumption of unsustainable resources (e.g., crude oil, coal, and natural gas), the global energy crisis has been much of an emergency (Kittner et al., 2017; Wang et al., 2018; Sadhukhan et al., 2018; Ng et al., 2021). Many fossil fuels are exhausting rapidly with high emission of exhaust gases (e.g., carbon dioxide, sulfur dioxide, and nitric oxides); simultaneously, social and environmental problems such as acid rain and greenhouse effect are becoming more and more serious (Zhang et al., 2015; Venkata Mohan et al., 2016; Zhao et al., 2018; Li et al., 2020). At present, the promising strategy for improving the current issues of multicarbon energy *via* developing and searching for an alternative to fossil-based fuels has garnered much attention (Li et al., 2019a; Li et al., 2019b; Zhang H. et al., 2019; Nabil et al., 2021; Meng et al., 2022). Bioorganic carbon substances acting as sustainable and renewable biomass resources have a promising prospect for constructing a carbon-neutral society because of their abundance and wide existence in biological organisms, including straw, wood, and cotton as agroforestry biomass (Tan J. et al., 2021; De et al., 2020; Danish and Ahmad., 2018; Gazi, 2019; Zhang et al., 2021). In recent decades, biomass resources have been validated to be used as renewable and sustainable biomass materials, which are capable of being a pioneer in the field of alleviating resource shortage (Li et al., 2013; Li et al., 2014; Liu et al., 2015; Tareen et al., 2019; Kang et al., 2020). Lignocellulosic biomass is the most abundant content of renewable and sustainable biomass materials, considering further a building block for biomass resources (Tian et al., 2018; Mahajan et al., 2020). Because of its abundance, diversity, and inexpressiveness in merits, lignocellulosic biomass has a promising prospect for replacing fossil-based fuels in refining industrial production (Sánchez et al., 2019; Shen et al., 2020; Haldar et al., 2021). In essence, lignocellulosic biomass is a complicated bio-based substance including cellulose (30–50 wt%), hemicelluloses (20–40 wt%), and lignin (10–20 wt%) (Figure 1) (Sarip et al., 2016; Silva et al., 2018; Zhao et al., 2020). Cellulose is a polymer of glucose with varying degrees of polymerization; hemicellulose is a heteropolymer of pentose and glucose linked by *β*-1, 4-glycosidic bonds; lignin mainly consists of amorphous aromatic macromolecules (Cao et al., 2019; Mansora et al., 2019; Yang et al., 2020). In recent years, tremendous practical applications for cellulose converted selectively into some high value-added chemicals through multistep promotions have been focused (Li et al., 2017; Li X. et al., 2018; Wang H. et al., 2019; Slak et al., 2022), for instance, the efficient conversion of cellulose into levulinic acid by using cellulase-mimetic mesoporous solid acid (Shen et al., 2017), selective conversion of bio-based hemicellulose prehydrolysate to high-value succinic acid (Dalli et al., 2017), one-pot chemoenzymatic transformation of furfuryl alcohol from biomass (Qin and He., 2020), catalytic pyrolysis of cellulose into furan by solid acid catalysts (Nb_2_O_5_, *γ*-Al_2_O_3_, ZSM-5, and TS-1) (Zhu et al., 2021; Huang X. et al., 2022), and one-pot production of 5-hydroxymethylfurfural from cellulose using Brønsted-type catalysts (Al-SBA-15) (Shirai et al., 2017). Moreover, in pharmaceutical preparation, microcrystalline cellulose is a commonly used drug excipient, which is good for active drug molecules to be absorbed in the small intestine (Gadge, 2020; Benabbas et al., 2021). So, cellulose is a well-recognized commercial chemical.

More significantly, 5-hydroxymethylfurfural (5-HMF), a high value-added chemical, is a selective dehydration product of monosaccharides (e.g., glucose and fructose) or acid-catalyzed dehydration/hydrolysis of cellulose, which can become a versatile bio-carbon platform compound for upgrading other valuable refining industrial chemicals (Heo et al., 2021; Meng et al., 2022). 5-HMF is essentially a furfural derivative bearing hydroxymethyl (–CH_2_OH) and aldehyde group (CH = O) distributed at 1, 4-positions of the furan ring, which can be responsible for upgrading 5-HMF into value-added chemicals via oxidation and/or reduction. In the commercial survey, the 5-HMF’s market share will be predicted to reach up to EUR 55 million in 2024 (MarketWatch, 2019). Hence, 5-HMF as a refining chemical product devotes a great contribution to sustainable biorefinery, and 5-HMF as a core stock presents a powerful tool in the synthesis of antihypertensive, antidepressant, antianxiety, and anti-inflammatory drugs (Espro et al., 2021).

For the past few years, selectively catalytic conversions of cellulose into refining high-value chemical of 5-HMF over various potent solid acids have been paid much more attention (Flores-Velázquez et al., 2020; Liu G. et al., 2022; Liu S. et al., 2022). Many efficient solid acids as catalysts with novel carbon frameworks have been developed and utilized in selective biomass conversions. The related investigations on some protocols for cellulose converted into 5-HMF loading heterogeneous solid acids have been only briefly documented and discussed in previous published reviews (Shen et al., 2020; Xu et al., 2020; Tempelman et al., 2021; Slak et al., 2022). In this review, numerous recent applications of the selective conversions for cellulose into 5-HMF as a high value-added industrial chemical are emphasized via heterogeneous catalysis. Moreover, solid acids as potent heterogeneous catalysts comprising Brønsted and/or Lewis acidic sites are discussed in affecting the selective conversion of cellulose into 5-HMF. Therefore, summarizing heterogeneous solid acid catalysts with novel frameworks enables us to further understand their role in the selective conversion of cellulose.

## 2 Cellulose Biomass

**FIGURE 2:**
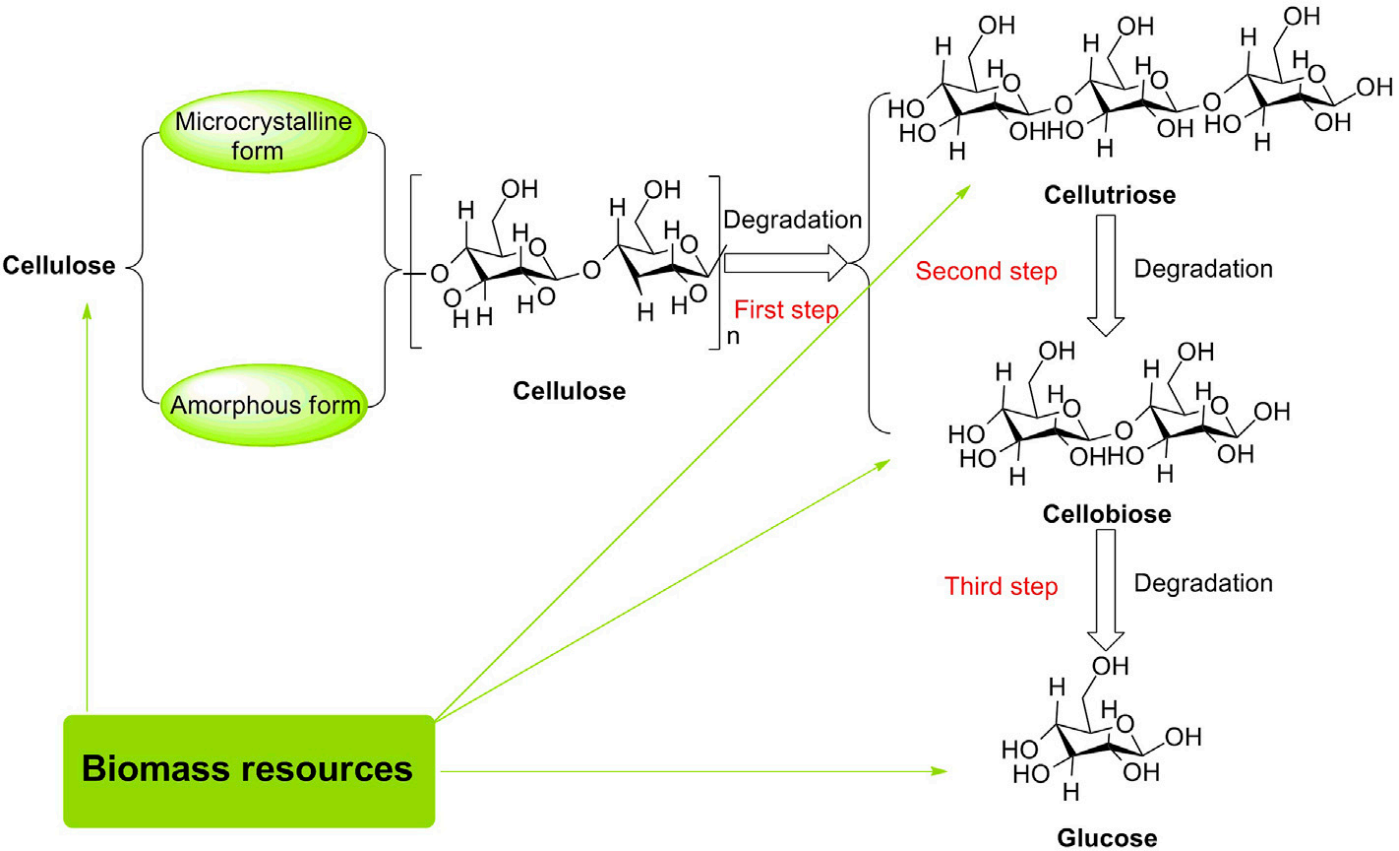
General route to selective biomass conversions of cellulose to glucose.

Cellulose, one of the major components of lignocellulosic biomass, is composed of D-glucose linked by *β*-1,4-glycosidic bonds, having 30–50 wt% in quantity (Qi et al., 2018; Zhao et al., 2020; Vanderfleet et al., 2021). Conventionally, cellulose has both microcrystalline and amorphous forms (Haan et al., 2007; Wang et al., 2021). Microcrystalline cellulose as a stable carbohydrate with a robust crystalline structure is not sensitive to acidic conditions, as compared to amorphous cellulose without a densely packed region that can form an ordered matrix. Herein, it is worthy to be noted that the formation of the ordered matrix for polysaccharides can effectively inhibit the contact of acids with 1, 4-glycosidic bonds in the bulk phase (Heinze, 2015; Tkachenko et al., 2021). For instance, the hydrolysis of microcrystalline cellulose into glucose in the presence of solid acids (e.g., sulfonated active carbon (AC-SO_3_H), magnetic solid acid (Fe_3_O_4_-SBA-SO_3_H), and macroporous resin Amberlyst 15) was just tolerated to be 40.5, 21, and 15% in yields of glucose, respectively (Onda et al., 2009; Lai et al., 2011). To the best of our knowledge, D-glucose is a product of complete hydrolysis of cellulose; additional hydrolysates of cellobiose and cellotriose are products of partially hydrolyzed cellulose (Guo et al., 2019; Lehrhofer et al., 2022). They are usually utilized as the main feedstock for the preparation of high value-added industrial chemicals because of multiple hydroxyl groups present in their molecular skeletons. These hydroxyl groups are reckoned as efficient reaction sites that enable interaction with Brønsted/Lewis acidic sites originating from potent solid acid catalysts (Figure 2). Therefore, the interaction between cellulose/cellobiose/cellotriose/D-glucose and acid catalysts is beneficial for the 1, 4-glycosidic bond/C-O bond to be broken down and hydroxyl groups to be dehydrated in biomass conversions.

## 3 5-Hydroxymethylfurfural and Its Reactivity

### 3.1 Overview of 5-Hydroxymethylfurfural

5-HMF is a selective transformation product of cellulose (polysaccharide) through consecutive acidic hydrolysis, isomerization, and dehydration, which, at the same time, has been awarded to be the promising candidate of the “top 10 + 4” bio-based key platform compounds by the U.S. Department of Energy because it is capable of being a bridge between biomass feedstock and value-added industrial chemicals (Hu et al., 2020; Thoma et al., 2020). 5-HMF is a five-membered heterocyclic compound comprising one furan ring as the parental skeleton and one hydroxymethyl and aldehyde group located at 2, 5 positions that is permitted to be converted efficiently into various high-quality fuels and value-added chemicals by the oxidation and/or reduction reactions for the hydroxymethyl and/or aldehyde group(s).

### 3.2 Reactivity of 5-Hydroxymethylfurfural

**FIGURE 3:**
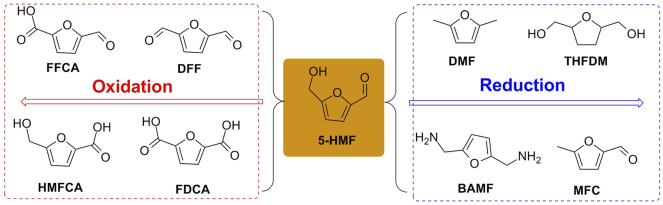
Transformation of 5-HMF into its derivatives.

The oxidation/reduction of 5-HMF can yield furanic derivatives such as 2, 5-furandicarboxylic acid (FDCA), 5-hydroxymethyl-2-furancarboxylic acid (HMFCA), 5-formyl-2-furancarboxylic acid (FFCA), 2, 5-diformylfuran (DFF), 2, 5-bis(aminomethyl)furan (BAMF), (tetrahydrofuran-2, 5-diyl)dimethanol (THFDM), 2, 5-dimethyl furan (DMF), and 5-methylfuran-2-carbaldehyde (MFC), which undergo oxidation/reduction of formyl and/or hydroxymethyl group(s) of 5-HMF to various extents (Figure 3).

#### 3.2.1 Reactivity for Oxidation

As taken, for example, the refining chemical of HMFCA was afforded through the selective aerobic oxidation for the formyl moiety of 5-HMF in the presence of Ag/ZrO_2_ catalysts, with more than 98% in yield (Schade et al., 2018). In the preparation of FFCA oxidized selectively from 5-HMF, CuO-CeO_2_, Ru/Al_2_O_3_, and MnO_2_–NaHCO_3_ mixed oxides were, respectively, used as a cheap and stable catalyst for catalytic conversion of 5-HMF into FFCA, and with the aid of diatomic oxygen as the oxidant in aqueous solution; the selectivity and conversion for FFCA showed good performance through the selective chemical oxidation (Ventura et al., 2016; Danielli da Fonseca Ferreira et al., 2018; Hayashi et al., 2019). Furthermore, the accessible approach to the selective oxidation of 5-HMF into DFF proceeded well by means of a Mn-based heterogeneous catalyst, with >99% selectivity and 100% conversion (Chen et al., 2019; Ke et al., 2019). As well, the photosensitive catalyst of WO_3_/g-C_3_N_4_ used as an electronic donator under visible light illumination could initiate catalytic oxidation of the 5-HFM solution at a certain concentration, retaining 87% in the selectivity of DFF (Wu et al., 2017). With a similar effect to photochemical oxidation, electrochemical oxidation becomes another available access from 5-HMF to FDCA. Bimetallic NiFe-layered double-hydroxide nanosheets loaded on carbon fiber paper and nickel boride decorated on the surface of Ni foam as electrodes were, respectively, testified to be potent in the electrochemical oxidation of 5-HMF into FDCA, with the yields of 99 and 98%, and excellent selectivity and conversions (Barwe et al., 2018; Liu et al., 2018). Based on the aforementioned experimental facts, it is definitely confirmed that the chemical oxidation of 5-HMF is indeed an effective gateway to the synthesis of further valuable refining chemicals in industrial production.

#### 3.2.2 Reactivity for Reduction

In terms of reduction for 5-HMF, the promising green solvent of DMF used as a “second-generation biofuel” is obtained by catalytic hydrogenation of 5-HMF (Rothamer and Jennings, 2012; Nishimura et al., 2014). In the investigation of direct hydrogenation of 5-HMF to DMF, a series of noble (Ru-, Pt-, and Pd-based) and non-noble (Ni- and Co-based) metal complexes are usually utilized to be potent Lewis acid sites in designing novel solid acids. In 2019, the reduction of 5-HMF into DMF was smoothly promoted by non-/noble metal-based solid acids, assisting hydrogen gas as a hydrogen source (Wang X. et al., 2019). In the same year, the Co-based metal catalyst of Co-(ZnO-ZnAl_2_O_4_) gave a 74% yield of DMF converted from 5-HMF (An S. et al., 2019). Simultaneously, the developed Co–graphene nanoparticle employed in the hydrogenation of 5-HMF to DMF was estimated definitely to be validated in the conversion of 5-HMF into DMF (Yang et al., 2019). Excitingly, 97% conversion of 5-HMF and 93% selectivity of DMF were successfully achieved by the utilization of the bimetallic Cu–Fe complex as a non-noble metal catalyst (Solanki and Rode, 2019). Significantly, the application of a non-noble bimetallic Cu–Ni electrode for the electrocatalytic reduction of 5-HMF into DMF was also in effect with the achievement of 91% conversion and 88% Faradic efficiency (Zhang Y. R. et al., 2019). Moreover, the commercial and valuable chemical BAMF prepared from 5-HMF as a feedstock was smooth to be on the run through the application of the combination of Ru/Nb_2_O_5_ and [Ru(CO)ClH(PPh_3_)_3_]/xantphos system (Komanoya et al., 2017). Afterward, the heterogeneous Ru-based catalyst reported by Pingen et al. was utilized for the one-pot amination of HMF to BAMF, exhibiting to be potent solid acid, with a 90% in yield of BAMF (Pingen et al., 2018).

All in all, the refining chemical of 5-HMF is a crucial building block for the synthesis of deeply high value-added chemicals in industry production and is also a significant selective catalyzed hydrolysis/dehydration product of cellulose. In recent years, the merits of 5-HMF as a feedstock utilized for upgrading high value-added industrial chemicals have appeared obviously in biomass conversions. However, the difficulty of selective conversion of cellulose into 5-HMF is still a necessity to be overcome, especially for the development of highly efficient catalysts for large-scale productions.

## 4 The General Pathway for Conversion of Cellulose Into 5-Hydroxymethylfurfural by Acid Catalysts

With the microcrystalline or amorphous form of cellulose coming up, it is usually utilized as a biomass raw material for the synthesis of various refining chemicals such as glucose, fructose, and 5-HMF. The selective conversion of cellulose into D-glucose is essentially a crucial step for other high valuable refining chemicals obtained from lignocellulosic biomass (Inoue et al., 2021; Yao, 2022). D-glucose as the complete hydrolysate of cellulose, with a six-membered ring framework, is flexible to be isomerized into a five-membered ring skeleton of fructose under acidic conditions. The intermediate of fructose bearing five hydroxyl groups in the conversion of cellulose can be transformed readily to 5-HMF by autocatalytic systems under heating surroundings. Certainly, the autocatalytic system in determining the conversion and selectivity of 5-HMF is closely related to the reaction temperature, being in direct proportion at a certain range. Nevertheless, the excessive temperature limits the conversion and selectivity of 5-HMF (Bocanegra et al., 2021; Lyu et al., 2022).

**SCHEME 1:**
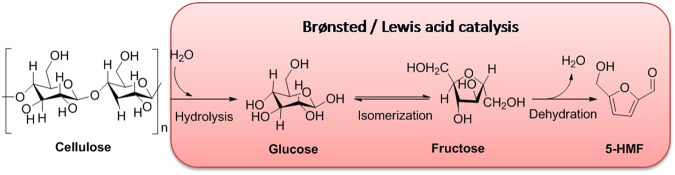
Plausible pathway of 5-HMF converted from cellulose by solid acids.

Catalysis is an effective strategy for producing refining industrial chemicals from biomass feedstock, which is able to lower the active energy of the reaction system and improve reaction conditions, as well as promote conversion and selectivity (Lange, 2021; Witzel et al., 2021). Catalysts acting as initiators of catalysis can be divided into homogeneous and heterogeneous coordinators (Bai et al., 2021; Taipabu et al., 2021). In the conversion of cellulose into 5-HMF, although homogeneous acid catalysts such as enzymes, mineral salts or acids, and supercritical water can overcome the resistance to degradation of cellulose for preparation of high-value chemicals, serious drawbacks for product separation, corrosion hazard, waste fluids, and severe reacting conditions are emerged obviously (Güell et al., 2015; Wang et al., 2021a; Chai et al., 2021). As compared to homogeneous acid catalysts, heterogeneous acid catalysts showing many merits, such as efficient conversion and selectivity, flexible separation, and low toxicity, have been extensively and popularly applied to industrial production and scientific research (Huo et al., 2015; Sun et al., 2016; Tan X. et al., 2021). To further understand the role of a heterogeneous acid catalyst in biomass conversions, exploring the mechanism of product 5-HMF converted from cellulose is favorable for novel catalyst design and improvement of hydrothermal conversion for biomass. As far as we know, a number of acid catalysts promoting the conversion of biomass are being on the same catalytic pathway involving Brønsted acid catalysis and Lewis acid catalysis (Scheme 1).

### 4.1 Brønsted Acid Catalysis

**SCHEME 2:**
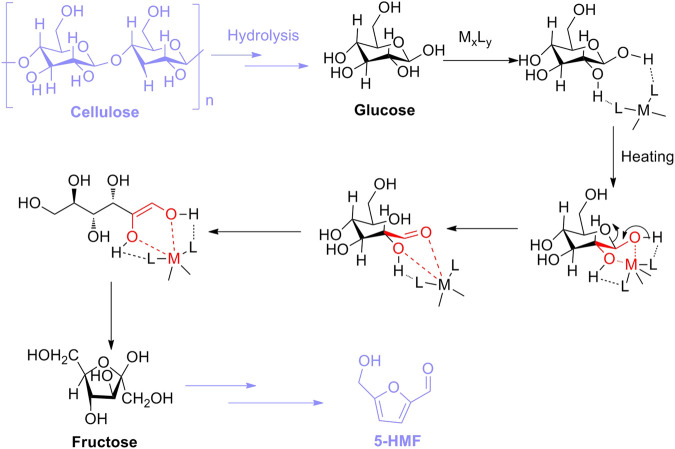
Brønsted acid-catalyzed hydrolysis mechanism of cellulose into 5-HMF.

In Brønsted acid catalysis, Brønsted acids are often considered to be potential catalysts in catalysis because of enabling the release of hydrogen ions (H^+^) in an aqueous solution via dissociation. H^+^ can protonate oxygen atom(s) in the 1, 4-glycosidic bond and/or pyranose ring. Subsequently, by undergoing the half-chair conformational isomerization of the oxygen atom neighboring C_1_ position of the anhydroglucose unit, it can bring about the formation of carbenium ion, presenting the cleavage of the C-O bond. Finally, in the presence of a water molecule, the carbenium ion interacted with the water molecule, enabling to reconstruct of the anomeric center to form D-glucose. After the formation of D-glucose, the isomerization of D-glucose into fructose occurs successively in acidic surroundings (Scheme 2) (Rinaldi and Schüth, 2009; Van Putten et al., 2013; Yamabe et al., 2013; Kang et al., 2018; Zhao et al., 2021). In this process, the critical intermediate of 1, 2-enediol is formed via a 1, 2-hydride shift that occurred in glucose. And then, as following steps of two 1, 2-eliminations, one 1, 4-elimination, and a ring closure in 1, 2-enediol, the promising production of 5-HMF is converted successfully from cellulose. Totally, the aforementioned pathway of 5-HMF converted from cellulose can be summarized by the following steps: 1) the protonation of oxygen atoms in the 1, 4-glycosidic bond and/or pyranose ring; 2) the cleavage of carbon–oxygen bond; 3) nucleophilic attack of water; 4) isomerization of glucose into fructose; and 5) successive dehydration (Zhao et al., 2021).

### 4.2 Lewis Acid Catalysis

**SCHEME 3:**
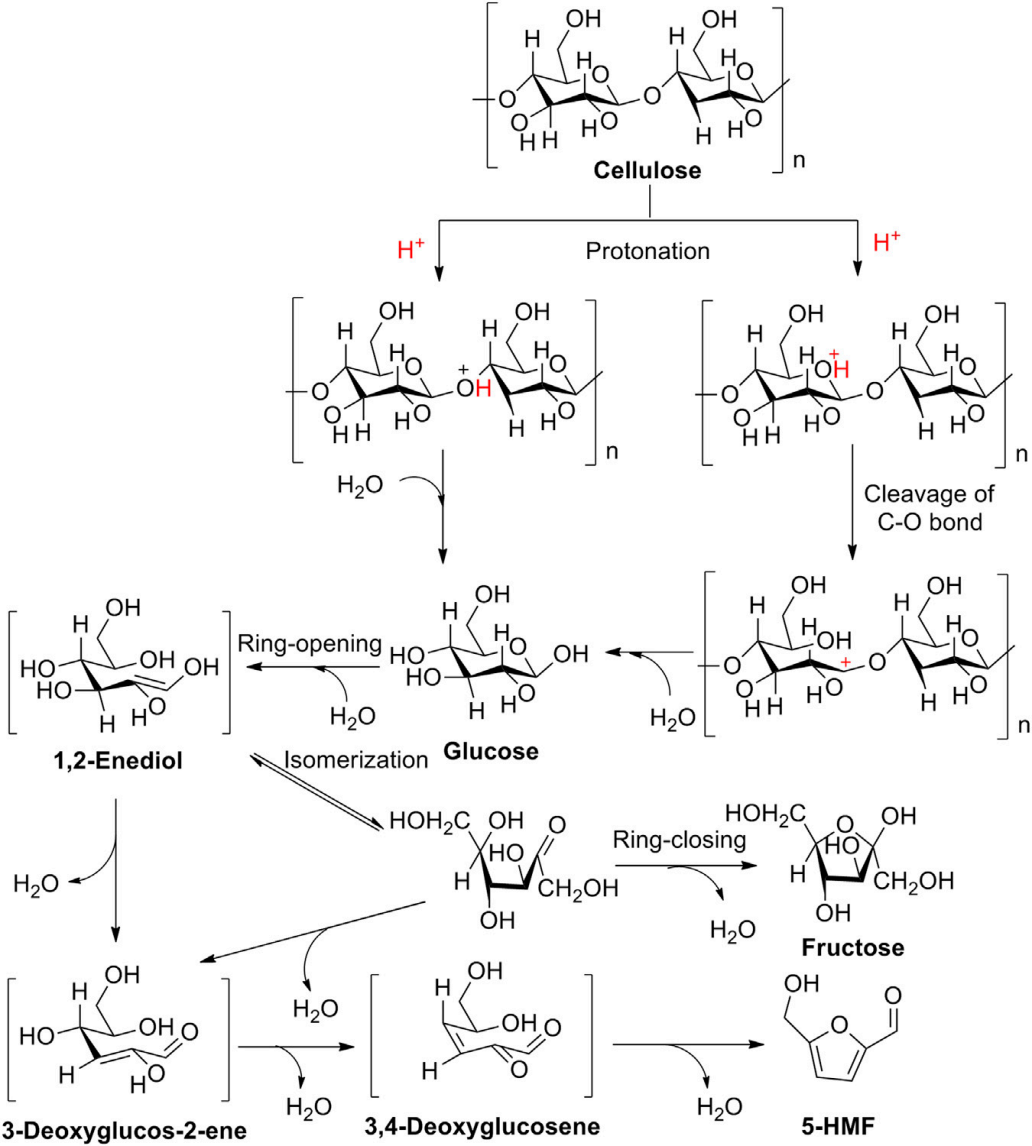
Lewis acid-catalyzed hydrolysis mechanism of cellulose into 5-HMF.

In comparison with Lewis acid catalysis, Lewis acids inhering empty orbits can accept electron pairs in chemical concepts (Marqués et al., 2021). In other words, acidic sites of Lewis acids are capable of being transformed into Brønsted acidic sites by combination with pairs of electrons donated from protic solvent, which can be responsible for the isomerization of glucose into fructose (Delidovich and Palkovits., 2015; Xu et al., 2017). In previous research studies, Lewis acidic sites of catalysts utilized for the aldose–ketose isomerization were conducted well because they could effectively promote the formation of enol intermediates (Charmot and Katz, 2010; Choudhary et al., 2013). Therefore, the general mechanism for Lewis acid-catalyzed isomerization can be summarized in two steps: one is the enolization of aldose, and the other is the 1, 2-hydride shift, both of which favor the isomerization of aldose into ketose. Moreover, in terms of glucose–fructose isomerization with Lewis acid, the isomerization promoted by Lewis acid (M_x_L_y_) should first follow the interaction of L of M_x_L_y_ with H of hydroxyl groups attached to the glucose skeleton. Afterward, under heating conditions, the formation of the five-membered complex occurs immediately, resulting from the coordination between M (Lewis acidic site) and two oxygen atoms (electron donors) in glucose. Then, the complex is authorized to be transformed into the corresponding enol intermediate through undergoing ring opening, which is subsequently isomerized into fructose. Eventually, as for the step of 5-HMF converted from fructose as an isomer of glucose, fructose is permitted to be directly dehydrated into 5-HMF with the assistance of the Brønsted acidic site (Scheme 3) (Hu et al., 2009; Zhao et al., 2021).

## 5 Applications of Acid Catalysts to Conversions of Cellulose Biomass Into 5-Hydroxymethylfurfural

Based on the aforementioned propositions of general pathways for conversions of cellulose into 5-HMF by Brønsted and Lewis acids, numerous heterogeneous acid catalysts with novel frameworks incorporating Brønsted and/or Lewis acidic sites have been emerging consecutively, such as sulfonated solid acid catalysts (Brønsted acidic sites), carbon-based acid catalysts (Brønsted acidic sites), M-zeolite acid catalysts (Lewis acidic sites), and heteropoly acid catalysts (Brønsted and/or Lewis acidic sites). Simultaneously, numerous achievements regarding biomass conversions have adequately convinced us that heterogeneous novel acid catalysts have a good promising prospect in biomass conversions.

### 5.1 Resin-Based Sulfonated Solid Acid Catalysts

**FIGURE 4:**
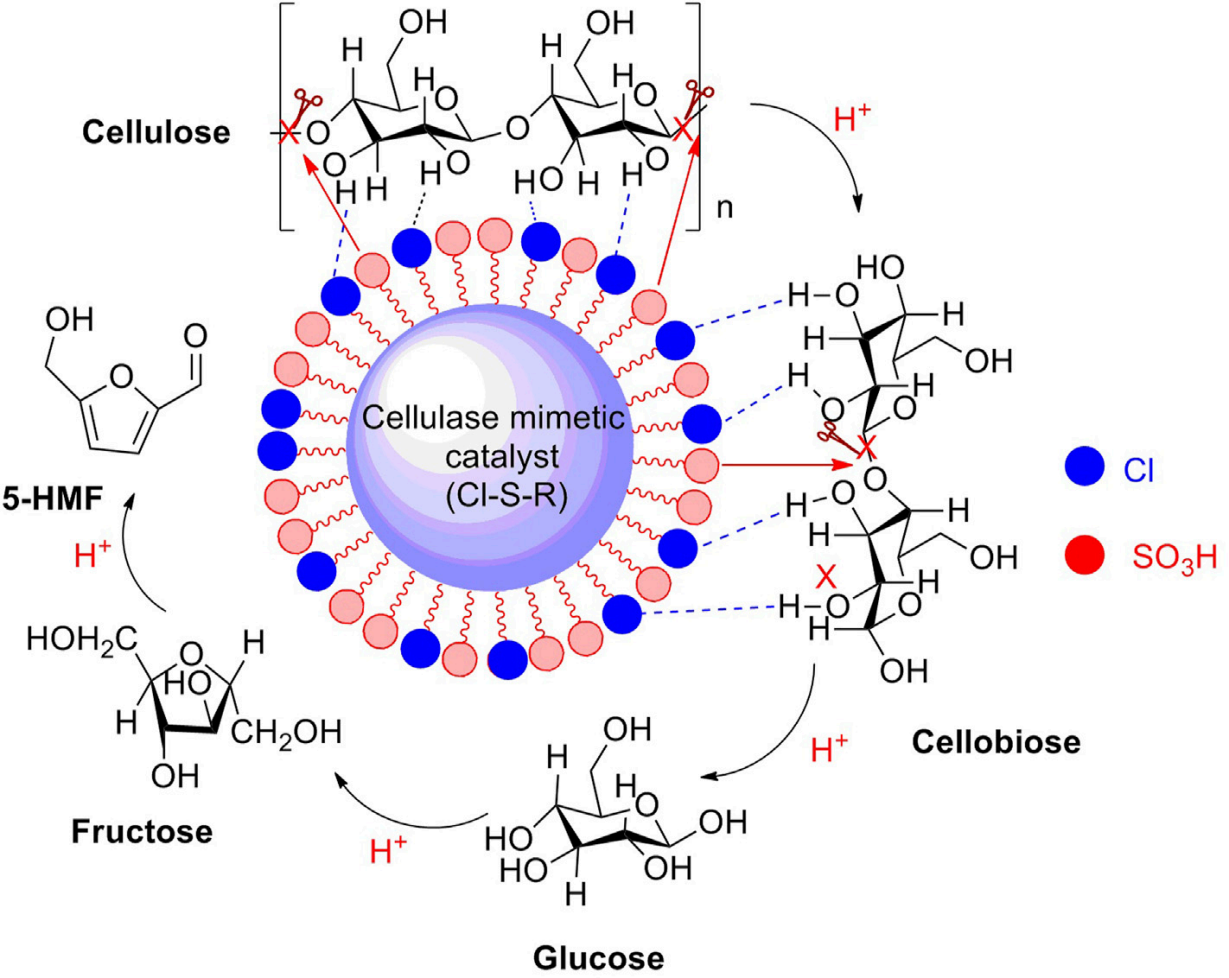
Route of cellulose converted into 5-HMF promoted by a solid acid catalyst (Cl_0.3_-S-R).

Resin-based sulfonated solid acids as potent heterogeneous catalysts have achieved good results in biomass conversions, resulting from the presence of sulfonic groups (-SO_3_H) on the frameworks of catalysts. The sulfonic groups are essentially Brønsted acidic sites, with strong acidity. In recent years, some novel resin-based sulfonated acid catalysts have been reported in the application of biomass conversions to high-value chemicals, with good results. The novel Cl-containing resin-based solid acid catalyst (Cl_0.3_-S-R) bearing with -Cl and -SO_3_H was synthesized by a simple hydrothermal method through the polymerization of *o*-chlorophenol and *p*-hydroxybenzenesulfonic with formaldehyde. Sulfonated solid acid with 1.47 mmol/g SO_3_H density on the external surface was thought to be an effective catalyst for corn stover conversion to 5-HMF and furfural production. Notably, cellulose is a major component of corn stover. In experimental results, the Cl-containing sulfonated acid catalyst exhibited a superior catalytic activity in the catalytic transformation of corn stover to 5-HMF in a 1,4-dioxane/H_2_O biphasic system, yielding a product of 5-HMF in a yield of 43.8% and the by-product of furfural in a yield of 38.1% (Figure 4) (Yang et al., 2021).

**FIGURE 5:**
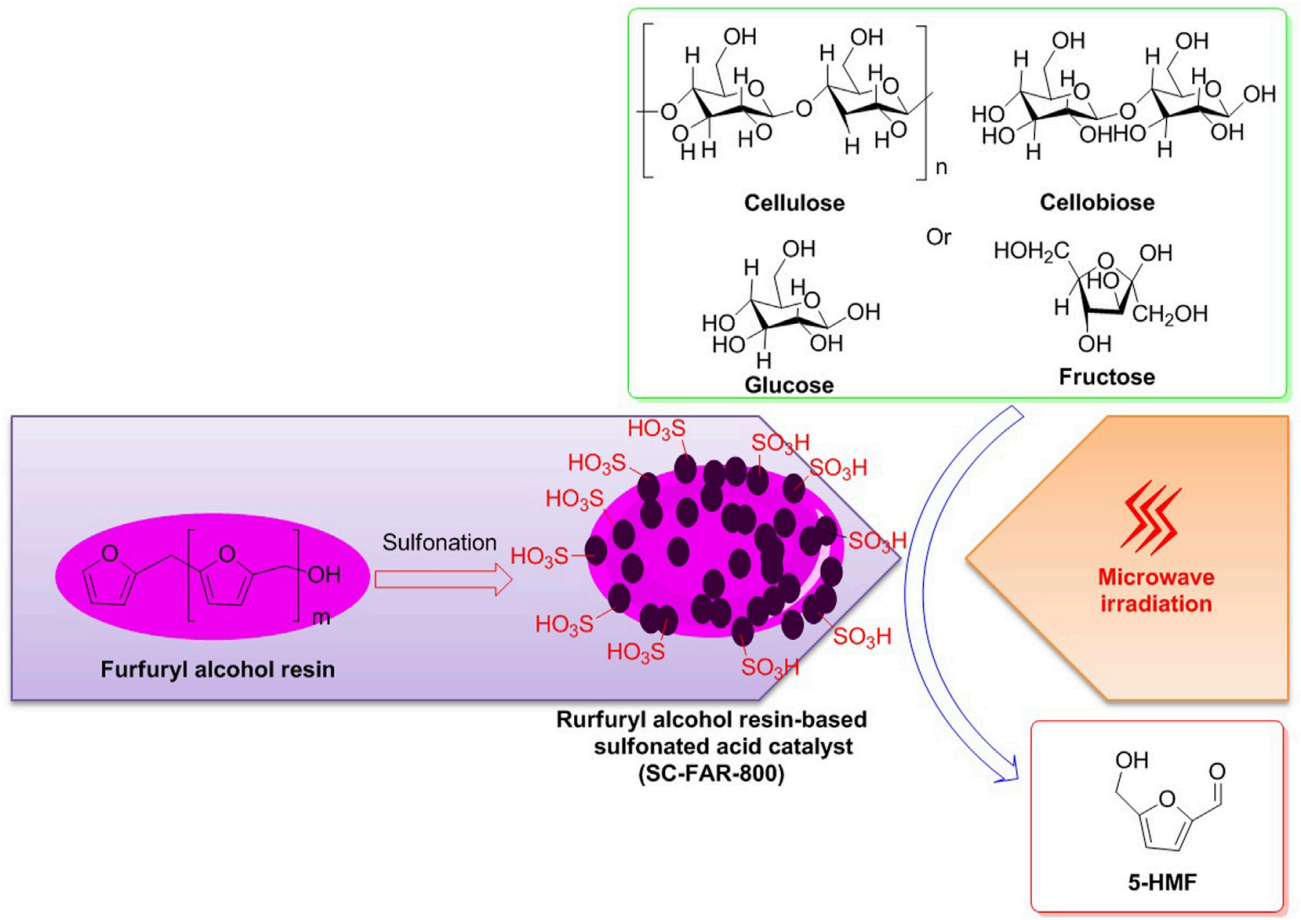
Catalyst SC-FAR-800 employed for biomass conversions into 5-HMF, with the assistance of microwave irradiation.

Afterward, with the assistance of microwave irradiation, employment of the catalyst SC-FAR-800 to the selective conversions of fructose, glucose, cellobiose, and cellulose into 5-HMF was exhibited to be in effect to varying extent, with the yields of 5-HMF being on 89.35, 38.17, 42.6, and 14.73%, respectively, indicating that fructose is a more appropriate feedstock for the synthesis of 5-HMF (Figure 5) (Huang T. et al., 2022). Moreover, the SC-FAR-800 is a furfuryl alcohol resin-based sulfonated acid catalyst, containing multiple sulfonic acid moieties with an acid density of 3.43 mmol/g. Moreover, by the techniques of SEM and BET, the irregular mesoporous structure of the sulfonated solid acid SC-FAR-800 was observed to be a specific surface area of 32.56 m^2^/g, an average pore size of 0.018 cm^3^/g, and a pore volume of 3.25 nm, which could be accountable for the efficient conversions of biomass.

### 5.2 Carbon-Based Acid Catalysts

**FIGURE 6:**
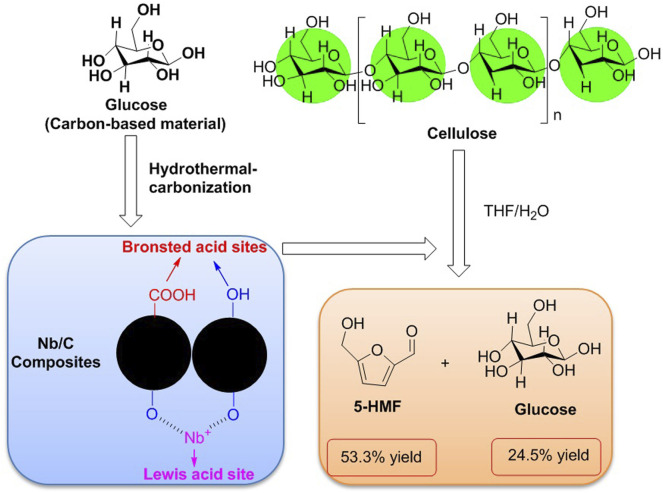
Hydrolysis and dehydration of cellulose promoted by Nb/C-50 as a carbon-based catalyst.

Carbon is the most basic element in bio-organism. Carbon-based materials such as activated carbon (AC), graphite, hydrothermal carbon, graphene, and carbon nanotube (CNT) have been in popularity in the design of novel functional carbon-based materials because carbon-based materials as potential supporters are capable to be in charge of the construction of novel frameworks. The novel Nb–carbon composite is a class of bifunctional carbon-based catalysts in possession of varying amounts of Brønsted and Lewis acid sites, which are able to be prepared by means of the hydrothermal carbonization method. Relying on the determination of catalytic activity for the hydrolysis and dehydration of cellulose, the agglomerated particle of Nb/C-50 can effectively enhance the selectivity of 5-HMF, which was achieved with a 53.3% yield of 5-HMF in a THF/H_2_O biphasic system at 170°C for 8 h (Figure 6) (Li Z. et al., 2018).

With the development of effective approaches to conversions of biomass, the eco-friendly and energy-efficient methods for cellulose conversion to 5-HMF were developed using modified activated carbon (AC) immobilized with metal ions. Activated carbon (AC) is a common carbon-based material. Via the treatment of diluted acid H_2_SO_4_, H_3_PO_4_, and HCl, it can, respectively, gain modified activated carbon supporters (ACS, ACP, and ACH). These supporters undergoing the immobilization of metal ions (M^+n^ = Cr^+3^, Fe^+3^, Cu^+2^, Zn^+2^, K^+^, and Al^+3^) in aqueous media were successfully transformed into carbon-based catalysts M-ACS/ACP/ACH. In the identification of catalytic activity, a potent metal carbon-based Cr-ACS catalyst was identified to be effective for 5-HMF conversion from cellulose, assisting ionic liquid [Bmim]Cl as a medium. Of course, it is emphasized here that immobilized metal ions can effectively improve Lewis acidic sites on the surface of catalysts (Tyagi et al., 2018).

**FIGURE 7:**
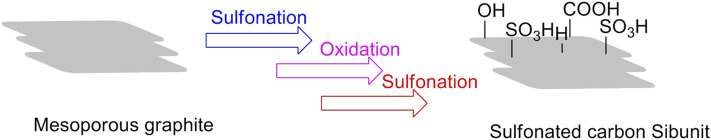
Sulfonated carbon Sibunit synthesized by using mesoporous graphite.

In the same year, the supporter of graphite-like mesoporous carbon material Sibunit had been documented by Gromov et al. (2018). Virtually, the supporter is also a carbon-based material. As a result of its inherent mesoporous properties, the carbon Sibunit treated by the following procedures of sulfonation, oxidation, and additional reaction for all the oxidized carbons sulfonated at 200°C was developed for the successive solubilization, hydrolysis, and dehydration of cellulose into 5-HMF in a one-pot reaction. The depolymerization of microcrystalline cellulose began at 180°C in water, in the presence of sulfonated carbon Sibunit solid acid. The main product 5-HMF could be achieved with the maximum yield of 22%, which was possibly ascribed to the formation of acidic groups (sulfuric, phenolic, and carboxylic groups) on the external surface of the carbon Sibunit (Figure 7) (Gromov et al., 2018).

Similarly, novel supporters of hydrothermal carbons (HTCs) as sugar-derived carbon materials prepared from monosaccharides are allowed to be obtained by suitable hydrothermal carbonizations (including reaction temperature and time). Subsequently, the resulting HTCs following the sulfonation can be transformed into potential novel carbon-based solid acids. The carbon-based acid (HTC220-6-SO_3_H) acting as a representative example was afforded through the hydrothermal carbonization of glucose for 6 h at 220°C, and subsequently the sulfonation for 15 h at 150°C. The HTC220-6-SO_3_H solid acid acting as a novel catalyst exhibited relatively high catalytic activity for the selective hydrolysis of cellulose and dehydration of fructose, indicating that sulfuric acidity (Brønsted acidity) can be responsible for the conversion to glucose and 5-HMF, with 43.63 and 20.29% yields of glucose and 5-HMF, respectively (Wataniyakula et al., 2018).

**FIGURE 8:**
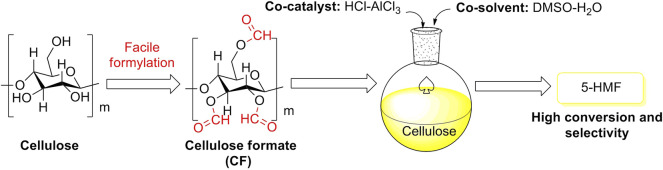
Selective conversions of cellulose biomass initiated by cellulose formate (CF) with the aid of co-catalyst and co-solvent.

Bio-carbon is one of the most promising carbon-based materials. Cellulose, acting as a representative bio-carbon, is usually considered to be a biomass raw feedstock for biomass conversions and an effective supporter for carbon-based catalysts. In recent studies, the supporter of cellulose *via* moderate formylation was facilely developed for cellulose formate (CF) production. The CF production with a special net-like structure and a high degree of formyl substitution was successfully applied to the selective conversions of cellulose biomass, assisting co-catalyst of HCl-AlCl_3_ and co-solvent of DMSO-H_2_O (Figure 8) (Jin et al., 2021). The exhibitions of conversion and selectivity to 5-HMF appeared to be excellent, as compared to monomeric glucose as another bio-carbon supporter (Table 1).

**TABLE 1:** Comparison of the cellulose to 5-HMF catalyzed by FeCl_3_, RuCl_3_, TiCl_3_, and VCl_3_ in the biphasic system (with or without NaCl in aqueous phase). (Jin et al., 2021)

Cat	Conditions	NaCl addition (%)	Organic phase	Cellulose conversion (%)	5-HMF selectivity (%)	5-HMF yield (%)
FeCl_3_	220C/20 min	35%	Butanol	97.3	75.2	73.2
		0	Butanol	97.3	62.0	60.3
		0	MIBK	97.3	55.3	53.8
		0	Hexanol	97.3	53.0	51.6
RuCl_3_	220C/30 min	35%	Butanol	95.2	87.5	83.3
		0	Butanol	95.2	67.8	64.6
		0	MIBK	95.2	64.6	61.4
		0	Hexanol	95.2	61.8	58.8
VCl_3_	220C/40 min	35%	Butanol	88.6	80.5	71.3
		0	Butanol	88.6	62.7	55.6
		0	MIBK	88.6	59.5	52.7
		0	Hexanol	88.6	56.9	50.4
TiCl_3_	220C/40 min	35%	Butanol	90.8	79.7	72.4
		0	Butanol	90.8	66.4	60.3
		0	MIBK	90.8	64.2	58.7
		0	Hexanol	90.8	61.9	56.2

Note: MIBK is methyl isobutyl ketone; catalyst loading: 0.125 mol/L.

### 5.3 Zeolite Catalysts

To the best of our knowledge, the framework of zeolite presents a promising constitution for a novel solid catalyst. In general, once non-/noble metal ions are inhered on the external surface of a zeolite structure, the catalytic activity of metal–zeolite material will be effectively improved, due to the enhancement of pore volumes and Lewis acidic sites on the external surface of the metal–zeolite occurred. In 2020, the access gateway of non-/noble metal ions (Cu^+2^ and Cr ^+3^) loaded on the external surface of ZSM-5 zeolite particles to the establishment of Cu-Cr-based zeolite was achieved by means of the ion exchange method. The highly crystalline Cu–Cr/ZSM-5 zeolite applied to the catalytic conversion of glucose into 5-HMF was smoothly initiated under suitable conditions, with good performance in conversion and selectivity (Chung et al., 2020).

Catalytic activity is always the key index for evaluating the performance of catalysts. Some classic zeolites of HY, Hβ, H-mordenite, and HZSM-5 as conventional solid catalysts are further discussed in catalytic activities for biomass conversions. In 2021, Zheng et al. (2021) reported on selective conversions of cellulose and starch over classic zeolites. The experimental results indicated that Hβ zeolite having appropriate Brønsted and Lewis acid sites becomes an effective promoter for furfural conversion from cellulose and starch. Simultaneously, HY zeolite with weak acidity is a flexible access to the transformation of 5-HMF from starch. H-mordenite and HZSM-5 zeolites bearing fewer Lewis acid sites on their external surfaces enable to inhibit the isomerization from glucose to fructose. On the whole, the generation of 5-HMF is closely correlated to the acid properties of zeolites. Simultaneously, it is confirmed that the acidity of zeolites can determine the target product formation.

**FIGURE 9:**
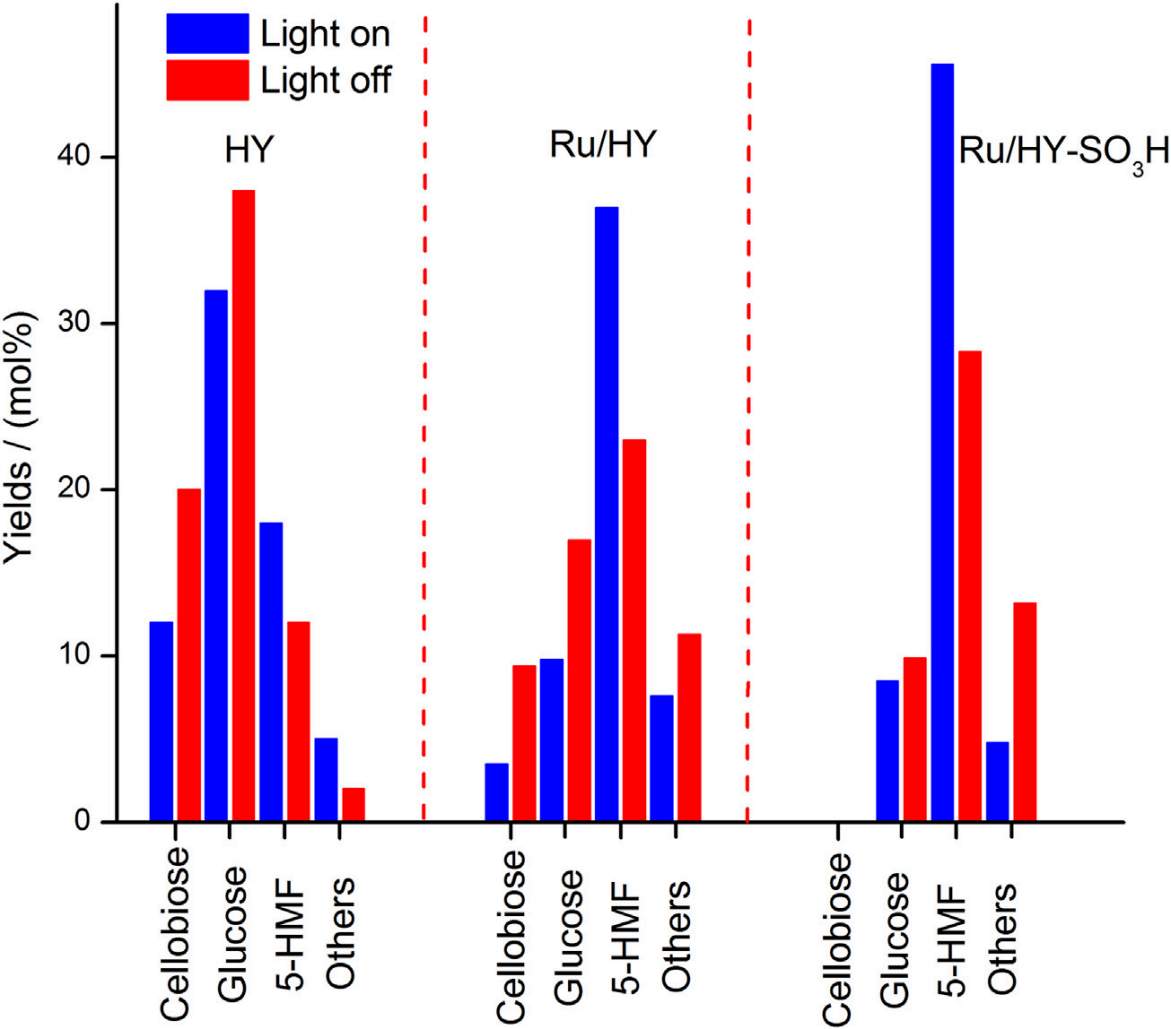
Performance on cellulose conversion over zeolite catalysts (Ru/HY-SO_3_H, Ru/HY, and HY) with/without light irradiation in a biphasic system.

Furthermore, the multifunctional zeolite catalyst (Ru/HY-SO_3_H) was developed by the introduction of Lewis and Brønsted acidic sites on the surface of zeolite for the selective cellulose conversion to 5-HMF, which was successfully prepared under metal immobilization and sulfonation (Wang et al., 2021b). Under light illumination and low temperature (120°C), the Ru/HY-SO_3_H, with the help of an ionic liquid/methyl isobutyl ketone biphasic medium, was capable of being a potent solid acid catalyst for the production of 5-HMF converted from cellulose. More significantly, the external surface of Ru/HY-SO_3_H irradiated by high-intensity light could be initiated on the plasmon resonance effect for the direct cellulose conversion, with a 48.4% yield of 5-HMF as selective production. Nevertheless, as compared to precursors of Ru/HY-SO_3_H, the promotions for selective conversion to 5-HMF by HY and Ru/HY were being on less in yields under the same conditions (Figure 9). It is implied that the formation of the plasmon resonance effect on the external surface of Ru/HY-SO_3_H can enhance more acidic sites for the selective conversion of microcrystalline cellulose.

Pham et al. (2020) disclosed a series of bifunctional sulfonated mesoporous silica materials used for the conversion of cellulose into 5-HMF in the Teflon-lined stainless steel reactor. These mesoporous silica catalysts (Zr-MCM-41) synthesized following the *in situ* method and sulfonation were given varied Brønsted acidic sites, which were identified by NH_3_-TPD analysis. Under heating for 2 h at 170°C, the sulfonated acid of S-15Zr-MCM-41 (73.44 wt% in silicon, 14.78 wt% in zirconium, and 11.78 wt% in sulfur) was allowed to be 70.2 and 16.4% in the conversion of cellulose and selectivity of 5-HMF, which were more than MCM-41 without sulfonation being on the conversion and selectivity of 15.2 and 1.3%.

### 5.4 Heteropoly Acids

**FIGURE 10:**
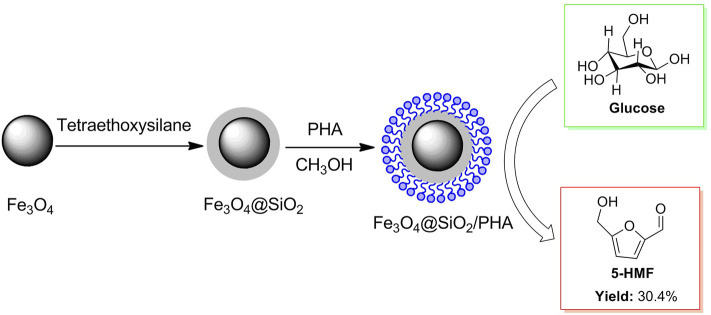
Synthesis and application of Fe_3_O_4_@SiO_2_/PHA in glucose conversion.

Heteropoly acids (HPAs) are a type of unique combination of hydrogen cations and polyoxometalate anions, which are composed of transition metal–oxygen anion clusters. Stable heteropoly acid (HPA) with strong Brønsted acidity and mild Lewis acidity is reckoned as an effective solid acid that is significant for the efficient conversion of renewable biomass to valuable chemicals. In 2015, a novel ionic crystal of metal-based HPA was emerged in the conversion of monosaccharides into 5-HMF. Cs_2_[Cr_3_O(OCC_2_H_5_)_6_(H_2_O)_3_]_2_[α-SiW_12_O_40_] acting as an HPA ionic crystal was witnessed to be a novel heterogeneous acid catalyst, with identifying components (W, 49.37; Cr, 6.83; Cs, 5.85; Si, 0.61 wt.%), in the dehydration of fructose or glucose into 5-HMF. The effects for fructose or glucose selectively dehydrated into 5-HMF were being on desirable results that were, respectively, 86 and 56% in yields of 5-HMF in DMSO media. In further investigation, the experimental results were demonstrated that lower Brønsted acidity of the HPA ionic crystal profits for the stabilization of 5-HMF with weak polarity, which may be a rational explanation for better catalytic performance of the HPA ionic crystal, as compared to conventional HPA (H_4_SiW_12_O_40_) (Yi et al., 2015). In 2020, a novel strategy for synthesizing a series of temperature-responsive HPA catalysts (Ch_n_H_5-n_CeW_12_O_40_, n = 1–5) was proposed by Lai et al. The Ce-based HPA of ChH_4_CeW_12_O_40_ was responsible for one-pot production of 5-HMF from cellulose, with the achievement of 67.5% in yield in a biphasic system (Figure 10) (Lai et al., 2020).

**SCHEME 4:**
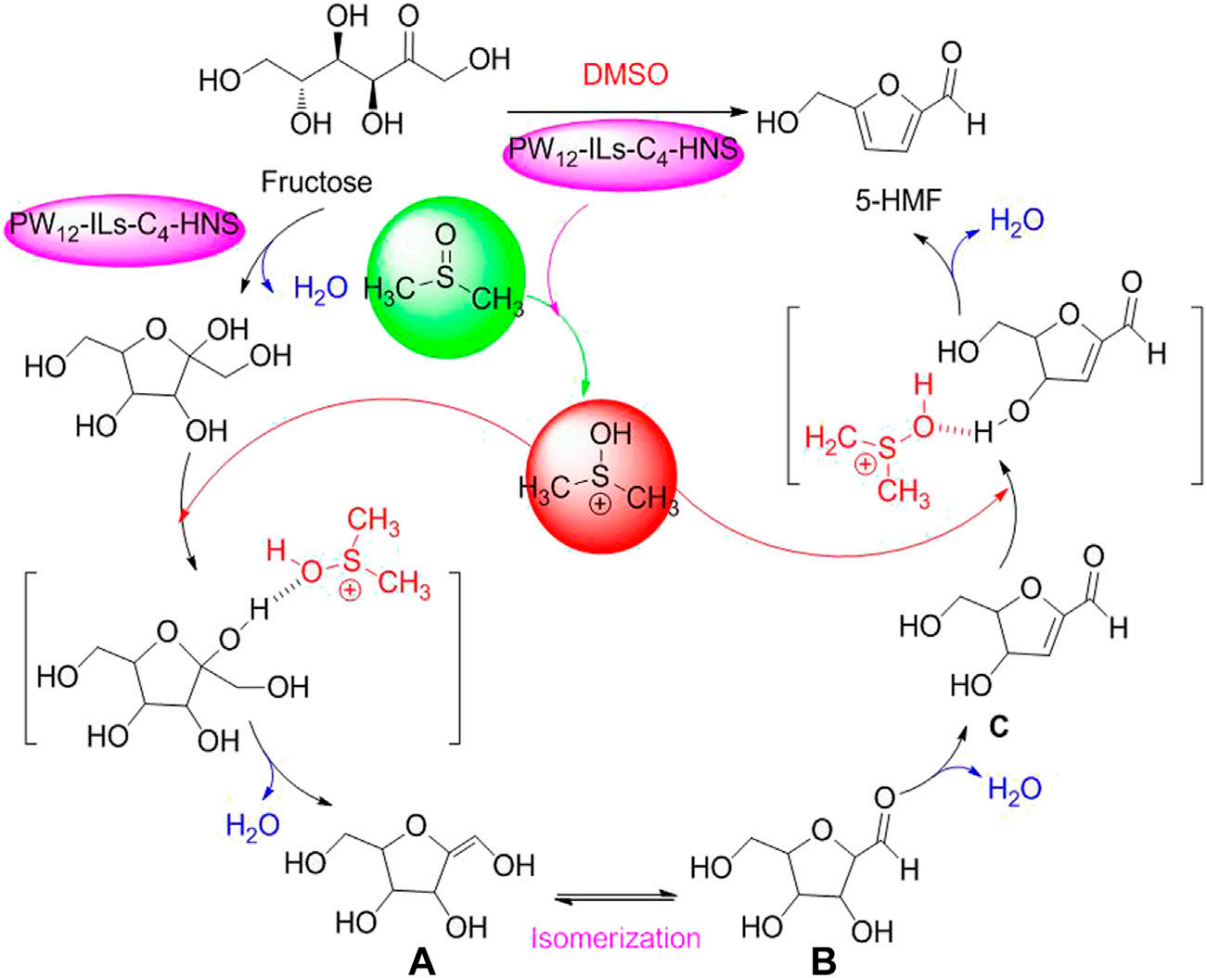
Proposed mechanism for selective conversion of fructose into 5HMF with the assistance of DMSO.

In addition, in terms of catalytic dehydration of fructose to 5-HMF by HPA, the newly plausible pathway for PW_12_-ILs-C_4_-HNS-catalyzed dehydration process of fructose to 5-HMF in DMSO was proposed by An Z. et al. (2019) (Scheme 4) (An Z. et al., 2019).

As shown in Scheme 3, the media molecule of DMSO is first activated by an active proton originating from PW_12_-ILs-C_4_-HNS. Then, the interactions between the activated DMSO and fructose happened through the formation of hydrogen bonds. With the release of H_2_O, the intermediate of enol **A** is formed. Enol **A** is transformed to corresponding aldehyde **B** (2, 5-anhydro-D-mannose) via isomerization. Afterward, the dehydrated product **C** is authorized to transform into 5-HMF via successive dehydration in the presence of PW_12_-ILs-C_4_-HNS and activated DMSO. PW_12_-ILs-C_4_-HNS as a novel PHA is a multicomponent solid acid, composed of phosphotungstic acid (H_3_PW_12_O_40_) and organosilica ((EtO)_3_ Si-ILs-C_4_) via immobilization to form corresponding organosilica hollow nanospheres. In terms of the organosilica hollow nanospheres, it was clearly figured out that H_3_PW_12_O_40_ of PW_12_-ILs-C_4_-HNS HPA is a Brønsted acid site, enabling strong electrostatic interactions with ILs. In practical biomass conversion application, the PW_12_-ILs-C_4_-HNS PHA exhibited excellent catalytic capacity and selectivity for acid-catalyzed dehydration of fructose to 5-HMF, under the conditions of DMSO as good media and heating for 2 h at 100°C. Moreover, the data of 93.7 % yield of 5-HMF and over six catalytic cycles were adequately witnessed that PW_12_-ILs-C_4_-HNS PHA is a cost-effective and environmentally benign catalyst (An S. et al., 2019). Similarly, glucose as a common monosaccharide, used as biomass feedstock to be upgraded into high value-added chemicals, has attracted extensive attention (Han et al., 2018; Li X. et al., 2018; Wei and Wu., 2018). The establishment of HPW-Nb_2_O_5_ calcined at 300°C to the selective conversion of glucose into 5-HMF was promptly documented in 2020 (Siqueira Mancilha Nogueira et al., 2020). The HPW-Nb_2_O_5_ was prepared through the combination of Nb_2_O_5_ (HY-340) as a support with H_3_PW_12_O_40_ as an active phase and subsequently calcination at 300°C. In optimizing reaction conditions, reaction temperature, reaction media, substrate concentration, and catalyst amount as essential factors were evaluated via Taguchi’s L_16_ experimental design. As a result, glucose at a concentration of 50g/L was allowed to be efficiently converted to 5-HMF (40.8% in yield), loading the HPW-Nb_2_O_5_ PHA (5%, w/v), with acetone–water media (1:1, v/v in ratio) at 160°C.

### 5.5 Other Solid Acids

In addition to aforementioned heterogeneous solid acids, other solid acids are still able to exhibit excellent catalytic activity for biomass conversions. The 2Al/SBA-15 catalyst containing 9.70 wt% in Al with a high amount of medium acid sites was permitted to be synthesized by means of the atomic implantation method. Because of the incorporation of appropriate Al amount into the framework of SBA-15, solid acid could present medium and strong acidic sites. Moreover, the catalytic activity and selectivity for the 2Al/SBA-15 were testified in the degradation of cotton cellulose to 5-HMF under mild hydrothermal conditions, with the performance of 2Al/SBA-15 in cellulose transformation with 5-HMF yield and selectivity of 68.5 and 62.1%, which was better than that of 3Al/SBA-15 (12.56 wt% in Al) with 46.03 and 64.49% in yield and selectivity of 5-HMF. The cause might be attributed to the formation of Al_2_O_3_ particles covering the acid sites created after the second Al layer deposition (Pham et al., 2019).

**FIGURE 11:**
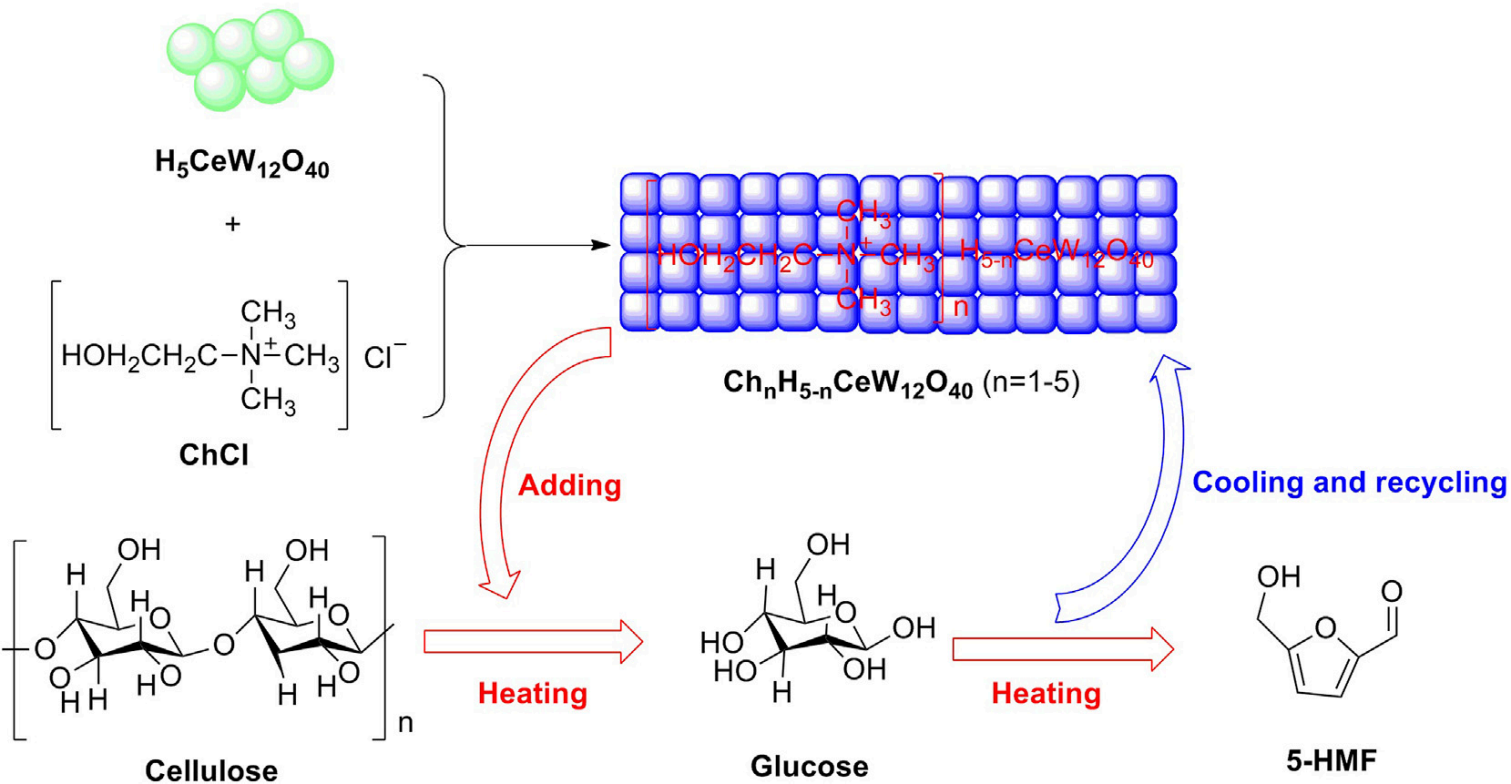
Ce-based HPA of ChH_4_CeW_12_O_40_ was synthesized and applied to the selective conversion of cellulose to 5-HMF under heating conditions.

D-glucose, a complete hydrolysate of cellulose, is usually used as biomass raw material to synthesize refining industrial substances in biomass conversions. Actually, the conversion of glucose into 5-HMF is a crucial intermediate step for cellulose being converted to 5-HMF. Considering the convenient separation of a catalyst from the mixture, a magnetic solid acid Fe_3_O_4_@SiO_2_/PHA was prepared by phosphotungstic acid (Brønsted acid) supported on magnetic nanoparticles (Fe_3_O_4_) coated by SiO_2_ (Figure 11) (Wang Y. et al., 2019). The magnetic solid acid in possession of good recovery by an external magnet could be on the run for four cycles in the glucose conversion to 5-HMF, without catalytic activity declining significantly.

In addition, heterogeneous catalysis asks for the catalyst and catalytic substrate to be in a varying phases and interacted on the external surface of the catalyst. Briefly, the catalyst is disabled to be dissolved in the solvent in the catalytic system. Therefore, inorganic metal salts are considered to be potential heterogeneous catalysts in the organic solvent. In 2019, an efficient strategy for one-pot conversion of microcrystalline cellulose into 5-HMF was introduced by the employment of non-/noble metal salts (e.g., FeCl_3_, RuCl_3_, VCl_3_, TiCl_3_, MoCl_3_, and CrCl_3_) in a biphasic system. The catalytic results demonstrated that RuCl_3_ as a potent catalyst exhibits excellent performance in the selectivity and conversion, with 83.3 and 87.5% in yield and selectivity of 5-HMF, in the NaCl aqueous/butanol biphasic system. Notably, the decrystallization and cleavage of 1, 4-glycosidic bonds in cellulose were able to be promoted through the interaction between transition metal chloride and cellulose, and subsequently, the consecutive isomerization, dehydration, and elimination of glucose being transformed into 5-HMF occurred (Yan et al., 2019).

In the same year, a hydrothermal solid acid (Yb_6_(BDC)_7_(OH)_4_(H_2_O)_4_) containing both bridging hydroxyls and metal-coordinated waters was introduced by Burnett et al. in glucose biomass conversions. The stable ytterbium metal–organic framework in possession of Brønsted and Lewis acid sites was considered to be a bifunctional catalyst, by which the conversion of glucose to 5-HMF was achieved successfully with 70% in selectivity in aqueous media (Burnett et al., 2019). With a similarity to the promotion of glucose into 5-HMF by Yb_6_(BDC)_7_(OH)_4_(H_2_O)_4_ in water, transition metal–oxide nanosheet aggregates, such as HNbWO_6_, HNb_3_O_8_, and HTiNbO_5_, were prepared by means of successive exfoliation and aggregation of layered metal oxides, which were good for yielding 5-HMF under hydrothermal conditions. In the conversion performance for 5-HMF, the catalytic capacities of these aggregated nanosheets were much better than that of ion-exchange resins and H-form zeolites under the same conditions. Meanwhile, it found that HNbWO_6_ nanosheets with an acid amount of 0.34 mmol/g exhibited higher selectivity for glucose conversion in the H_2_O–toluene biphasic system than fructose conversion (Takagaki, 2019). As a developing catalytic pathway of biomass conversion, a catalytic fast pyrolysis of cellulose biomass yielding value-added platform chemical 5-HMF was initiated over zirconium–tin mixed metal oxides (ZrO_2_–SnO_2_). The ZrO_2_–SnO_2_ metal oxide with 15 wt% Zr loading was mainly in charge of the increase in 5-HMF yield and selectivity, in a catalyst-to-cellulose ratio of 2/1, at 350°C as a pyrolysis temperature. In testifying the catalytic scope of ZrO_2_-SnO_2_-15 as a catalyst, various saccharides, such as cellobiose, maltose, glucose, and mannose, used as biomass stocks for selective conversion to 5-HMF were, respectively, evaluated under the same conditions. The conversion results indicated that the effects for the 5-HMF yield of glucose and mannose are slightly different, with 5-HMF yields of 12.16 wt% and 11.07 wt%. Maltose and cellobiose are deemed to be a dimer of amylose and cellulose, including, respectively, *α*- and *β*-O-4 glycosidic bonds. In their selectively catalytic pyrolysis, the yield of selective product 5-HMF from maltose and cellobiose were, respectively, 14.63 wt% and 14.21 wt% over ZrO_2_-SnO_2_-15 catalysts, which may be more conductive for *α*- and *β*-O-4 glycosidic bonds (Li Y. et al., 2021).

## 6 Conclusion and Outlook

More efficient biomass conversions into various refining chemicals become a hot topic of lower carbon energy regeneration and sustainability. Cellulose is the most abundant bio-based component in lignocellulosic biomass, with both microcrystalline and amorphous forms. Microcrystalline cellulose with a robust crystalline structure composed of *β*-1, 4-glycosidic bonds of D-glucose is limited to be automatically hydrolyzed into simple monosaccharides (e.g., glucose and fructose) in an aqueous solution. So, the degradation of cellulose becomes a difficulty in biomass conversions. High value-added chemicals (e.g., 5-HMF, THF, furfuryl alcohol, and levulinic acid) are the focus of renewable and sustainable biomass conversions all the time. The industrial production of 5-HMF acting as a monosaccharide derivative becomes a building block for biomass conversion, which can be responsible for a bridge between cellulose biomass and deeply refining high-value chemicals (e.g., FDCA, HMFCA, FFCA, DFF, BAMF, THFDM, DMF, and MFC). At present, the efficient strategy for selective conversion of cellulose biomass into 5-HMF should be attributed to acid-catalyzed dehydration/hydrolysis of cellulose in the presence of a potent acid catalyst. In this review, we summarize and discuss some catalytic strategies for acid-catalyzed dehydration/hydrolysis of cellulose into 5-HMF over varying novel solid acids. On the basis of Brønsted acid catalysis/Lewis acid catalysis, the strategies for selective conversion of cellulose into 5-HMF loading varying novel solid acids are categorized into the following: (I) employment of sulfonated solid acid catalysts to the selective conversion of cellulose biomass into 5-HMF, (II) carbon-based acids with novel frameworks utilized to the synthesis of value-added chemical of 5-HMF, (III) 5-HMF converted from cellulose occurred on the external surface of zeolite catalysts, and (IV) selective acid-catalyzed transformation into 5-HMF from cellulose biomass by other heterogeneous solid acids. Totally, powerful solid acid as a heterogeneous catalyst employed for the selective conversion of cellulose biomass into 5-HMF is undoubtedly effective access to biomass conversions in the laboratory.

To the best of our knowledge, the reactivity of 5-HMF has been well recognized by chemists. The functional groups (-CH_2_OH and C=O) located on the furan ring of 5-HMF are facilitated to be transformed into expected moieties to afford corresponding high value-added chemicals, via selectively catalytic oxidation/reduction. Nevertheless, it requires well that related oxidant/reductant with powerful selectivity and catalytic capacity are well developed. 5-HMF is widely regarded as a promising candidate for well-known bio-based platform compounds. Relying on its functional groups’ oxidation/reduction, various furfuryl derivatives are permitted to be afforded to support the development of renewable and sustainable biomass resources. Therefore, in the current multicarbon energy crisis, 5-HMF is capable of being a pioneer for developing lower carbon energy. In future works involving biomass conversion, considering complicated components of raw biomass resources, designing a well-tolerated/-selective heterogeneous acid catalyst is still a necessity for biomass conversions with high selectivity on large-scale production.

In summary, the objective of this review is to disclose available strategies for 5-HMF selectively converted from cellulose biomass, opportunities and challenges faced by various solid acid catalysts in the heterogeneous catalysis system and to provide reactivity of 5-HMF (oxidation and reduction) upgrading into varying refining high-value chemicals and some Brønsted/Lewis acid catalysis theory for guiding future biomass development.
